# School and learning contexts during the COVID-19 pandemic: Implications for child and youth mental health

**DOI:** 10.1007/s12144-022-03941-y

**Published:** 2022-11-24

**Authors:** Kimberley C. Tsujimoto, Katherine Tombeau Cost, Kaitlyn LaForge-MacKenzie, Evdokia Anagnostou, Catherine S. Birken, Alice Charach, Suneeta Monga, Elizabeth Kelly, Rob Nicolson, Stelios Georgiadis, Nicole Lee, Konstantin Osokin, Paul Arnold, Russell Schachar, Christie Burton, Jennifer Crosbie, Daphne J. Korczak

**Affiliations:** 1 Department of Psychiatry, Hospital for Sick Children, 1145 Burton Wing, 555 University Ave, Toronto, ON M5G 1X8 Canada; 2 Department of Psychiatry, Faculty of Medicine, University of Toronto, Toronto, ON Canada; 3 Deprtment of Pediatrics, Faculty of Medicine, University of Toronto, Toronto, ON Canada; 4 Holland Bloorview Research Institute, Toronto, ON Canada; 5 Division of Pediatric Medicine, Hospital for Sick Children, Toronto, ON Canada; 6 Departments of Psychology and Psychiatry, Queens University, Kingston, ON Canada; 7 Department of Child and Adolescent Psychiatry, University of Western Ontario, London, ON Canada; 8 Department of Psychiatry & Behavioural Neurosciences, McMaster University, Hamilton, ON Canada; 9 Toronto District School Board, Toronto, ON Canada; 10 Department of Psychiatry, University of Calgary, AB Calgary, Canada; 11 Department Medical Genetics, University of Calgary, AB Calgary, Canada

**Keywords:** Mental health, School format, Covid-19, School-aged, Children, Adolescents

## Abstract

Despite significant disruption to school during the COVID-19 pandemic, research on the impact on children is sparse. This study examines in-person and virtual learning contexts and the impact of school format on mental health (MH). Children and adolescents were recruited from community and clinical settings. Parents and children completed prospective online surveys about school experiences (November 2020) and MH symptoms (February/March 2021), including school format and activities. Standardized measures of depression, anxiety, inattention, and hyperactivity were collected. Hierarchical regression analyses tested associations between school format and MH. Children (*N* = 1011; aged 6–18 years) attending school in-person (*n* = 549) engaged in high levels of participation in COVID-19 health measures and low levels of social learning activities. Learning online in high school was associated with greater MH symptoms (*B* = -2.22, CI[-4.32,-.12] to *B* = -8.18, CI[-15.59,-.77]). Children with no previous MH condition that attended school virtually experienced a similar magnitude of MH symptoms as those with previous MH conditions. However, children who attended school in a hybrid in-person format, with no previous MH condition, experienced less hyperactivity as same-age peers with prior MH problems (*B* = -8.08, CI[1.58,14.58]). Children’s learning environments looked very different compared to before the pandemic. Removing children from school environments and limiting opportunities that support their MH, such as social learning activities, is problematic. Efforts to address the learning contexts to protect the mental health of children are needed.

## Introduction

The SARS-CoV-2 (COVID-19) pandemic has led to major disruptions to the daily lives of children, youth, and their families (de Miranda et al., 2020; Loades et al., 2020; Nikolaidis, et al., 2021). One of the greatest disruptions has been to the school environment (Lee, 2020). During the pandemic children and youth have had to adjust to new ways of learning, including virtual learning, due to school closures. Even when schools were open to in-person learning, the enforcement of public health measures resulted in a school climate that looked very different from that of previous years (see Government of Ontario’s Guide to Reopening Schools, 2020). Bronfenbrenner’s well-known ecological systems theory (Bronfenbrenner & Morris, 1998) posits that a child’s development is influenced by their relationships with surrounding environments. This framework is helpful in conceptualizing the way in which a child’s school environment may impact their development in a related, but different domain such as their mental health (MH) and well-being. During the pandemic, young people have been asked to adjust to a changing school environment, including the addition of public health measures (e.g., Carbon, 2020), which may be contributors to declining MH and emotional wellbeing (Cost et al., 2022; Racine et al., 2021). However, necessary adjustments have extended beyond changes to the physical environment (Lee, 2020; Verlenden et al., 2021).

The Centre for Disease Control (CDC) has reported on the effects of attending school online versus in-person during the pandemic. The results showed that online learning was associated with the presence of psychosocial stressors (e.g., child mental and physical well-being and parents’ emotional distress), and was greatest for those who attended school completely online, followed by those in a mixed format (i.e., partially online, partially in-person), and then those who attended completely in-person (Verlenden et al., 2021). The CDC report relied on parents’ reports of school, which limits the understanding of children’s perspectives of their school experiences during the pandemic. In addition, the study did not report on the activities that children were participating in while attending school online or in-person. A description of children’s learning environments during the pandemic is currently underreported in the literature, making it difficult to understand school-related changes.

Children in Ontario, the most populous province in Canada, were subjected to repeated and prolonged school closures during the 2020–2021 academic year as a result of public health directives (Government of Ontario’s Guide to Reopening Schools, 2020). During school closures, children attended school online. At other times of the year, parents had the opportunity to choose to send their children to school in-person to varying degrees. As children continue through a third disrupted school year (2022–2023), it is important to examine the impact of their school experiences on MH outcomes. It is critical to understand the changes to the school environment that are associated with effects on MH. Research on the impact of school format during the pandemic and the changes to the school context more generally, are understudied, which leaves the association between pandemic disruption to school and MH unclear. It is possible that while some changes to children’s learning are challenging for their MH, some may support their needs both in and out of the context of the pandemic (Roy et al., 2022). Research examining these associations is critical to ensure that children and youth have a positive school experience moving forward, not only for their learning success but also for their mental wellbeing.

Research during the first year of the pandemic reported worsening mental health (MH) of children and youth (Cost et al., 2022; Panda et al., 2021; Ravens-Sieberer et al., 2022), with 25% and 20% of youth reaching clinically significant levels of depression and anxiety, respectively (Racine et al., 2021). One recommendation to remediate deteriorating MH is to provide safe opportunities for children and youth to have social interactions (Cost et al., 2022) . However, ongoing restrictions and repeated lockdowns make this difficult to achieve. Despite periods of re-opening during more recent pandemic phases, emerging research shows that the burden of child and youth depression and anxiety has persisted over the course of the first year of the pandemic (Fancourt et al., 2021; Li et al., 2021; Liu et al., 2022).

As children and youth spend a large proportion of their day at school, it is important that they are offered engaging and meaningful opportunities to learn not just academically, but also personally and socially. School settings allow young people to develop a sense of self, competency, and agency, while also offering opportunities to form relationships; all of which are positive enablers of good MH (Jerusalem & Hessling, 2009; Riekie et al., 2017; Shochet et al., 2006). Moreover, when students’ MH needs are met, they are more likely to be successful academically (O’Connor et al., 2019).

The current study reports on children’s school experiences during the 2020–2021 school year. The primary objective was to compare MH outcomes (depression, anxiety, inattention, hyperactivity) across in-person and virtual school formats. The selected MH outcomes span internalizing and externalizing domains in pediatric populations. Secondarily, we sought (1) to report on the activities that children have engaged in during school hours and (2) to determine whether some students’ MH may have been disproportionately impacted by the changes to school. To date few studies have examined the direct association between different school formats and mental health across the full range of school ages. In addition, descriptions of each school format are limited and to date, children’s perspectives have been largely ignored. This study addresses these gaps by testing associations between school format and mental health from kindergarten to grade 12 and reports on both child and parent descriptions of in-person and virtual learning environments during the pandemic.

## Methods

### Participants and procedures

The sample included children and adolescents participating in an ongoing, longitudinal study investigating the impact of the COVID-19 pandemic on child and youth mental health. Children and youth were recruited from clinical and community settings. Clinic-recruited participants (Outpatient Psychiatry, Province of Ontario Neurodevelopmental Disorder (POND) network; POND, 2020) included children and adolescents aged 6 to 18 years referred to outpatient mental health clinics for mental health concerns including, but not limited to, depression and anxiety disorders, attention-deficit-hyperactivity disorder (ADHD), obsessive compulsive disorder (OCD), disruptive behavior disorders, autism spectrum disorders (ASD), and intellectual disability. Community-recruited participants included children aged 6 to 18 years recruited at an urban science museum in Toronto as part of a population-based community research sample (Spit for Science, 2020). Clinical and community samples were previously established cohorts with a diverse existing child and adolescent participant base for investigations into the impact of the COVID-19 pandemic on child and youth mental health (AUTHOR CITATION). Parents who had previously consented for future contact were sent an invitation to participant in the present study. Their children, aged 10 years or older, were sent a separate link to participate if they were interested.

An electronic survey (using the survey application REDcap; Harris et al., 2009) was used to collect the data and was first sent in May 2020, with an ongoing recruitment design with bi-annual follow-up. Parents who previously consented to be contacted for research were emailed an invitation to participate in the present study and sent a separate link to give their child if the child also wanted to participate. Children were considered eligible to participate if they were 10 to 18 years old, due to the measurement design, which used electronic surveys. Data on school variables were collected in November 2020; data on MH outcomes were collected in February/March 2021. All participants who completed the school survey (November 2020) were considered for analyses in this study. This research was approved by all institutional research ethics boards; participants provided informed consent and/or assent.

### Study context: Grade stratification and school format

School format in the present study is defined by the primary mode of instruction at time of data collection. At the time of data collection regarding school format (November 2020) the Ontario provincial government offered different options for attending school. Parents of children enrolled in elementary and middle school (K-8) were given the choice to attend school virtually/remotely *or* in-person with enforced health protocols (discussed below). Adolescents enrolled in high school, grades 9 to 12, were able to attend school either fully virtually/remotely, *or* in a mixed format (some days in-person and other days virtually). High school students were not able to attend school fully in-person. Further details about the provincially enforced decisions for attending school during the time of the study (2020/2021) are available elsewhere (Ministry of Education, 2020). In addition, during the study period, the province had experienced periods of school closures in January and February 2021. Therefore, while some participants opted for in-person or mixed school formats at the start of the school year, some may have been required to attend school virtually due to province-wide closures.

### Measures

#### Exposures: School context

School context and learning experiences of children during the COVID-19 pandemic was assessed using an electronic self-report questionnaire. Parents reported on their school-aged children (5 to 18 years; junior kindergarten to secondary school). In addition to parents’ reports, children aged 10 years and older reported on their own experiences during the pandemic, including learning activities and attitudes towards school. For in-person school, participants were asked to report on the frequency of child engagement on 13 items including: sitting individually, group work, 1:1 time between students and teachers, and several public health measures. These public health measures included: wearing masks indoors (i.e., hallways and classrooms), physical distancing in hallways, avoiding sharing and playing games, avoiding crowds and gatherings, self-isolating when symptoms are present, washing and sanitizing hands, and avoiding physical contact such as hugs and high fives. All items were rated on a Likert-type scale with 5 degrees of endorsement: 1 = never, 2 = rarely, 3 = sometimes, 4 = often, and 5 = always. Good reliability was achieved for child (α = 0.80) and parent (α = 0.87) reports of the in-person school context and learning experiences.

#### Outcomes: Child and youth mental health

##### Depression

The Revised Child Anxiety and Depression Scales-Parent Version (RCADS-P; Ebesutani, et al., 2015), is a widely used measure with good reliability for our study age group (see Korczak et al., 2022). The Major Depressive Disorder (MDD) subscale includes 10 items rated from 0 to 3 (0 = “never”, to 3 = “always”). Total scores were converted into standardized *T* scores, which allow for a more appropriate interpretation of depressive symptoms relative to a child or adolescent’s sex and age. Greater scores indicate greater levels of depression with a *T* score ≥ 65 indicating clinically significant symptoms (Ebesutani et al., 2015).

##### Anxiety

Anxiety symptoms were assessed using the Screen for Child Anxiety Related Disorders (SCARED), parent report (Birmaher et al., 1997) which is a widely used measure with good reliability for our study age group (see Korczak et al., 2022). Items describe feelings or experiences and are rated from 0 to 2 (0 = “not true” or “hardly ever true”, to 2 = “very true” or “often true”) for a possible total score ranging from 0 to 82, with greater scores indicating greater levels of anxiety. This study reported on the 9-item Generalized Anxiety Disorder subscale with total scores of 9 or greater indicating clinically significant levels of anxiety.

##### ADHD

The Strengths and Weaknesses of Attention-Deficit/Hyperactivity Disorder (SWAN; Swanson et al., 2012) is a widely used measure with good reliability for our study age group (see Korczak et al., 2022). The parent report included two subscales, for inattention and hyperactivity. Each subscale was made up of 9 items that are rated from – 3 = “far below average” to + 3 = “far above average”. A continuous total score was calculated for inattention and hyperactivity and adjusted so that greater scores indicated greater levels of ADHD symptoms.

#### Covariates

Data on child age, ethnicity, sex, household income, and pre-COVID MH diagnosis (MH or neurodevelopmental disorder) were gathered using an adapted version of the CRISIS questionnaire (Nikolaidis et al., 2021); a measure designed to assess mental health during the COVID-19 pandemic.

Descriptive statistics examined engagement in social learning activities and public health measures from kindergarten to grade 12, across school formats: in-person, virtual, and mixed. Analyses were stratified by grade (K-8 and 9–12) due to differences in school format options described above. School format was used as a grouping variable across analyses. Child demographic data including sex, ethnicity, household income, and pre-pandemic MH diagnosis were entered as covariates in each analysis. All data met assumptions for linear regression. When data were complete on school grade, missingness was minimal across independent variables (0%-3.6%), except for household income (20.1%). When multiple siblings were enrolled, the sibling with more complete data was included. When missing data did not differ between siblings, the data for the older sibling were selected to increase the total number of youth-reports of school since self-reports were only available for children ≥ 10 years. Longitudinal analyses with the same population and/or during the same time period (Fancourt et al., 2021; Li et al., 2021; Liu et al., 2022) showed that participant MH outcomes were stable over time during the pandemic. Therefore, when available (< 5% across all MH outcomes from K-12), missing MH data were imputed using data from the previous time point.

Descriptive analyses were reported on complete data only and parent and child reports were examined separately. Hierarchical regression analyses tested associations between school format and MH. These analyses were run with and without imputed data and results were replicated. In block 1 of each regression model, children’s demographics including sex assigned at birth, ethnicity, and household income were entered. In block 2 children’s MH history and school format were entered as independent variables. In the final block, an interaction between MH history and school format was entered. The standardized measures for MH outcomes were analyzed for the age groups for which they have demonstrated validity. Therefore, younger children (< grade 3) were excluded from analyses with standardized MH outcomes. Data were cleaned and analyzed with RStudio version 4.1.0 (Team. R. Studio, 2020) and SPSS version 27.0 (IBM Corp, 2020).

## Results

### Demographics

The sample (*N* = 1011) included children from kindergarten to grade 12 (K-12) and provided good representation across developmental school ages in primary (grades K-3; *n* = 245); junior/middle (grades 4–8; *n* = 520) and high school (grades 9–12; *n* = 253). Participant demographics are presented in Table 1. Participants were an average of 10.39 years (SD = 3.31) of age, and equally represented across males and females. Over half of the sample identified their ethnicity as European and of those who reported their household income, just over half were from greater socio-economic backgrounds. Just under 30% of school-aged children had been identified with a mental health condition before the pandemic.

**Table 1:** Participant Characteristics and School Format During the COVID-19 Pandemic

	Elementary School (Grades K-8)	High School (Grades 9–12)
	*N* = 799	*N* = 253
Sex (Male proportion)	440 (55.1%)	112 (44.3%)
Child ethnicity (European ancestry proportion)	485 (60.7%)	161 (63.6%)
Annual Household income (> $79,999 Cdn)	462 (57.8%)	139 (54.9%)
Mental health diagnosis (pre-pandemic)^a^	216 (27.0%)	90 (35.6%)
Depression	20 (2.5%)	35 (13.8%)
Anxiety	155 (19.4%)	73 (28.9%)
Attention-Deficit/Hyperactivity Disorder	95 (11.9%)	21 (8.3%)
Obsessive Compulsive Disorder	27 (3.4%)	15 (5.9%)
Learning Disability	51 (6.4%)	21 (8.3%)
Autism Spectrum Disorder	19 (2.4%)	8 (3.2%)
School format (in-person)^b^	549 (68.7%)	–-
School format (mixed)^b^	–-	179 (70.8%)
School format (virtual)^b^	217 (27.2%)	67 (26.5%)

^a^Children were considered to have a pre-existing mental health (MH) diagnosis pre-pandemic if they had a diagnosis history of one or more of the following conditions or neurodevelopmental disorders: anxiety, depression, obsessive compulsive disorder, learning disorder, attention-deficit/hyperactivity disorder, autism spectrum disorder. Using this criteria, many children had comorbid mental health diagnoses.

^b^School format varied by age/grade. When schools were open to in-person learning, families of children in elementary and middle schools were given the choice to attend in-person or virtually from home, while high school choices were mixed format (in-person and virtual) or completely virtually.

### School format: How did children attend school?

When schools were open to in-person learning, most participants in elementary school attended school in-person (68.7%), but just over one quarter (27.2%) attended virtually (Table 1). Without the choice to attend fully in-person, most participants in high school attended school in a mixed format (70.8%) with a combination of in-person and virtual learning from home, and 26.5% reported attending school only virtually. In elementary school (grades K-8), no differences in school format were observed when comparing males and females (*χ*^2^(1) = 0.07, *p* = 0.80) or children with and without pre-pandemic MH diagnoses (*χ*^2^(1) = 0.29, *p* = 0.65). Similarly in high school (grades 9–12), no differences in school format were observed when comparing males and females, (*χ*^2^(1) = 0.21, *p* = 0.66) or children with and without pre-pandemic MH diagnoses, (*χ*^2^(1) = 0.02, *p* = 0.96).

#### What did in-person learning look like?

##### Elementary and middle school grades K-8

**Fig. 1:**
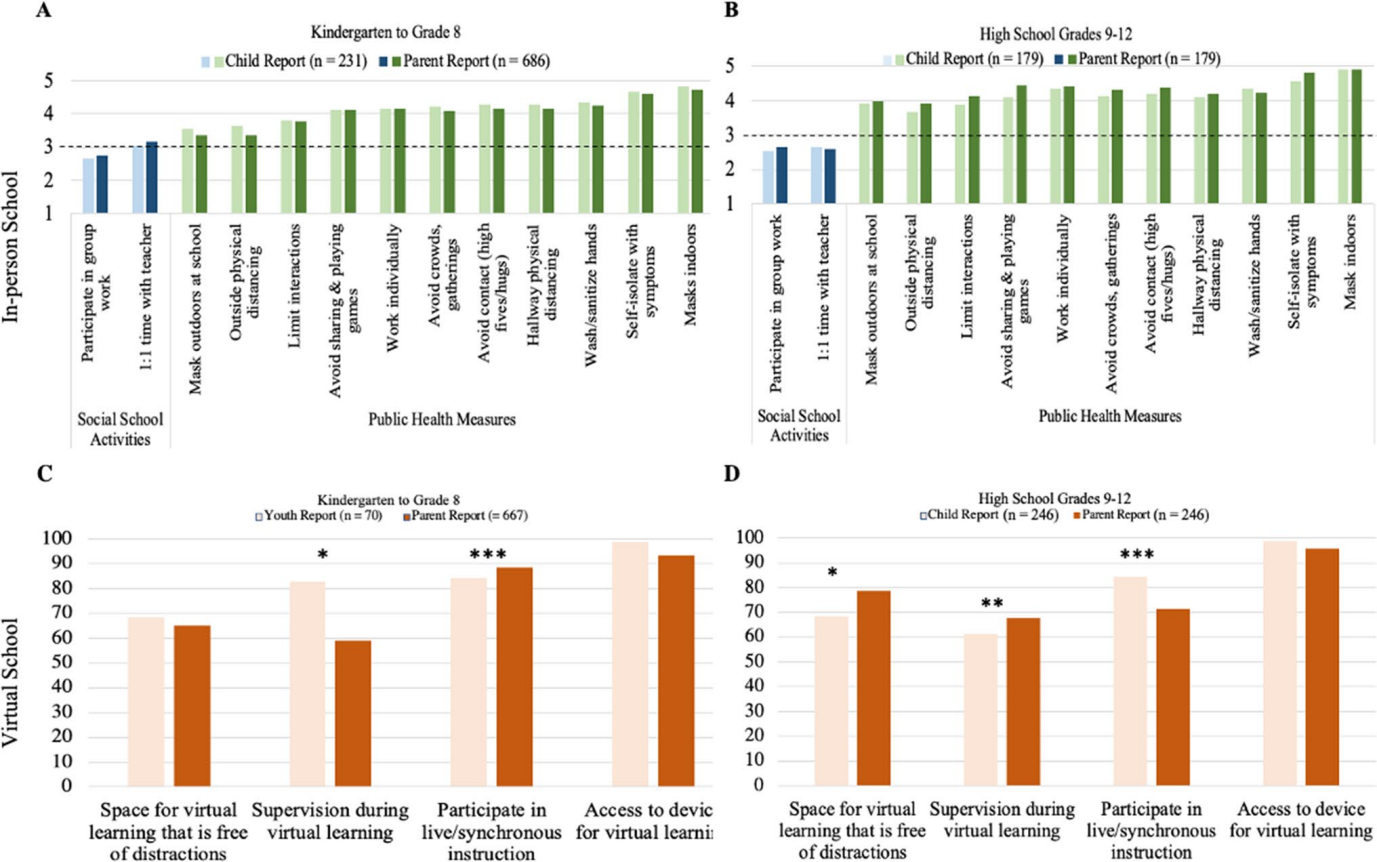
Child and parent reports about in-person and virtual school experiences during the COVID-19 pandemic. Note. Lighter shades represent youth reports and darker shades represent parent reports. The y-axis for in-person school (**A, B**) represents a 5-point Likert-type scale ranging from 1 = never to 5 = always. Social learning activities are shown in blue and public health measures in green. In-person school activities that fall below the dotted line occur rarely to never. Results for virtual learning (**C, D**) from home are reported as percentages (y-axis) with greater values indicating greater endorsement. Asterisks indicated significant differences between parent and child reports. *p < 0.05, **p < 0.01, *** p < *0.001*

When attending school in-person, parents and their children reported “often” or “always” engaging in public health measures (*M*_range_ = 3.78 to 4.74; Fig. 1). Public health measures for outdoor activities at school were reported as occurring slightly less frequently, including outdoor physical distancing (*M* = 3.35, *SD* = 1.14) and wearing masks outdoors while at school (*M* = 3.35, *SD* = 1.29). With respect to classroom learning, individual seated work occurred “very often” (*M* = 4.16, *SD* = 0.85), while more social learning activities such as group work and one-on-one time between teachers and students occurred “rarely” or “sometimes” (*M* = 2.75, *SD* = 1.04 and *M* = 3.15, *SD* = 0.94, respectively). Parents and their children reported similarly for each in-person learning activity (Fig. 1, panel A).

##### High school grades 9–12

When attending school-in person, families and their children in high school reported “often” or “always” participating in public health measures (*M*_*range*_ = 3.89 to 4.92). Results also showed that in-person high school learning involved often working individually at desks (*M* = 4.36, *SD* = 0.75) with minimal participation in group work (*M* = 2.52, *SD* = 1.06) or one-to-one time between students and teachers occurring only “sometimes” or “rarely” (*M* = 2.64, *SD* = 0.96). Public health measures for outdoor activities were reported as occurring slightly less frequently, including physical distancing (*M* = 3.68, *SD* = 1.11) and wearing masks outdoors at school (*M* = 3.93, *SD* = 1.18). Parents and their children reported similarly for each in-person learning activity (Fig. 1, panel B).

#### What did virtual learning look like?

##### Elementary and middle school grades K-8

For virtual learning, significant differences between parent and child-report were found for supervision during online learning from home [χ^2^(1,70) = 8.31, *p* =  < 0.05], with greater endorsement from children (82.9%) compared with parents (58.9%). Significant differences were also found for whether online learning included live/synchronous instruction [χ^2^(1,70) = 30.91, *p* =  < 0.001], with greater endorsement from parents (88.6%), compared with children (84.3%). No significant differences between parent and child report were found for access to a quiet, distraction-free space for online learning [χ^2^(1,70) = 2.67, *p* = 0.13]. Almost all children (98.6%) who completed the survey about online learning reported having a device for online learning, and most parents reported similarly (93.3%, Fig. 1, panel C).

##### High school grades 9–12

For virtual learning, significant differences between parent and child-report were found for having quiet, distraction-free space for online learning at home [χ^2^(1,253) = 5.74, *p* < 0.05], with greater endorsement from parents (78.7%), compared with their children (68.3%). Significant differences were also found for parent supervision during online learning [χ^2^(1,253) = 10.69, *p* < 0.01] with greater endorsement from parents (67.7%), compared with their children (61.2%). Finally, significant differences were found for whether virtual school included live/synchronous instruction; [χ^2^(1,253) = 16.05 (1), *p* < 0.001], with greater endorsement from children (84.3%), compared with their parents (71.6%). There was no significant difference between parent and child reported access to a device for virtual learning [χ^2^(1,253) = 0.01, *p* = 0.99] with > 90% endorsement for each (Fig. 1, panel D).

### School format and mental health outcomes

#### Junior elementary and middle school: grades 4–8

##### Depression

**Fig. 2:**
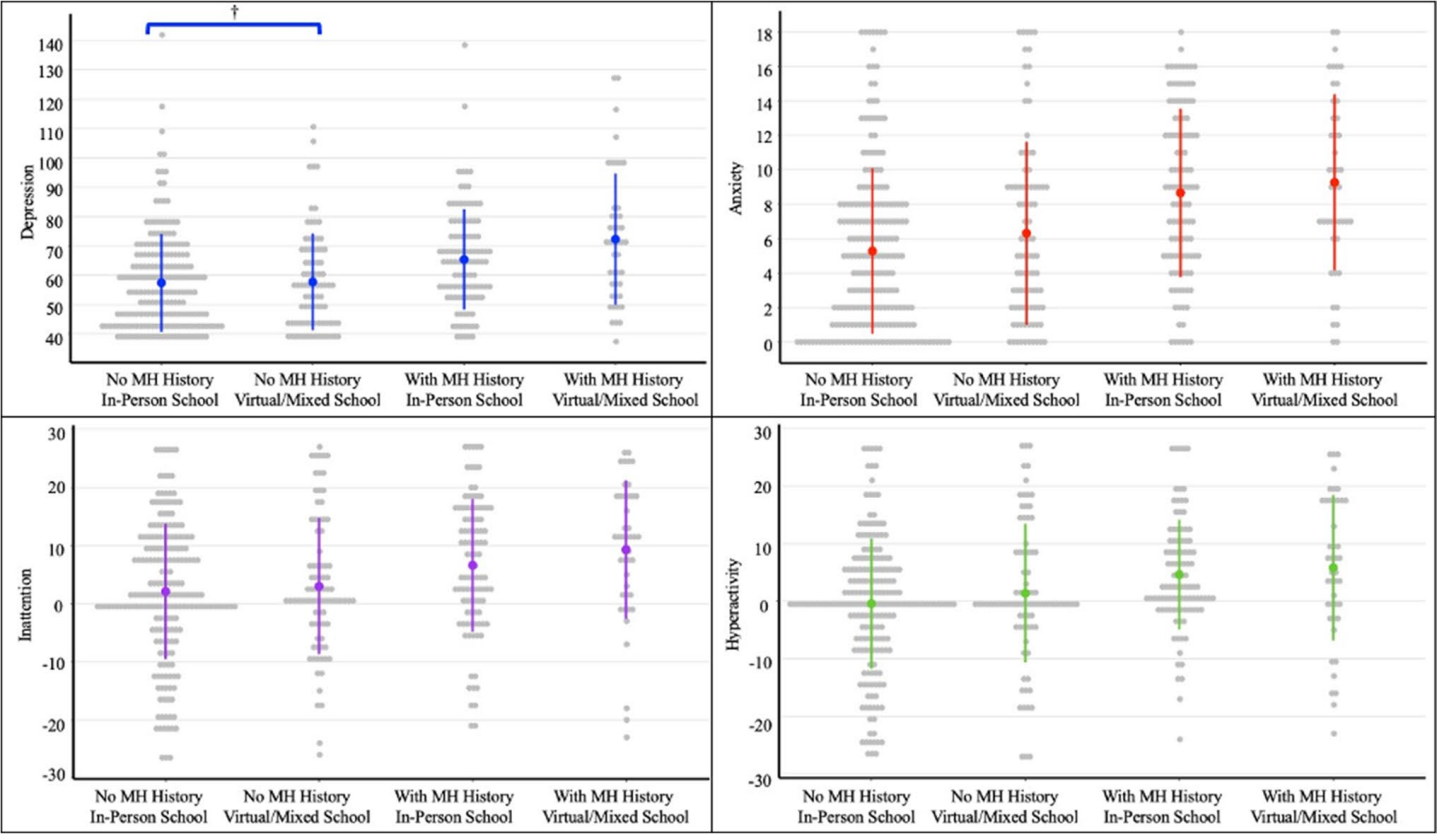
School Format and Mental Health Symptoms among Elementary School Children (grades 4 to 8) with and Without a History of pre-COVID Mental Health Problem (*n* = 535). Note. Grey dots represent total values for each MH outcome, with greater values indicating greater MH symptoms (i.e., worse MH). For inattention and hyperactivity, a score of zero represents typical symptom levels. The colored dots represent mean values for each group (MH x School Format) with error bars represented by colored lines. Brackets above groups with “No MH History” identify trending (†*p* < 0.10) interaction effects between school format and pre-pandemic MH history

School format was not associated with later depression, however, the interaction with MH history trended towards significance (B = 6.85, SE = 3.77; *p* = 0.07), with greater depression symptoms among children with a previous MH diagnosis attending virtually. Children’s depression was significantly associated with sex (B = -5.47 SE = 1.68; *p* < 0.01) and ethnicity (B = -3.59, SE = 1.73; *p* < . 05). Greater symptoms of depression were found for males and children identified with European ancestry. Household income was not associated with later depression (B = 3.74, SE = 2.14, *p* = 0.10). Having a previous MH diagnosis was associated with greater depression later (B = 9.19, SE = 1.74, *p* < 0.001) (Fig. 2; Appenidx Table 2).

##### Anxiety

The association between school format and anxiety symptoms trended towards significance (B = 1.01, SE = 0.51, *p* = 0.05) suggesting greater anxiety among those who attended school virtually. Children’s ethnicity was associated with later anxiety (B = -2.04, SE = 0.50, *p* < 0.001), specifically children identified with European ancestry had greater symptoms of anxiety. Sex and household income were not associated with later anxiety (B = -0.58, SE = 0.48, *p* = 0.19; B = 3.74, SE = 2.14, *p* = 0.32, respectively). Having a previous MH diagnosis was associated with greater anxiety later (B = 3.02, SE = 0.49, *p* < 0.001). There was no interaction effect for MH history and school format (Fig. 2; Appendix Table 3).

##### Inattention

Sex was significantly associated with later inattention (B = -5.80, SE = 1.11, *p* < 0.001) with greater inattention among males. Ethnicity trended towards significance, suggesting greater inattention among children identified with European ancestry (B = -2.09, SE – 1.15, *p* = 0.06). Income was not associated with later inattention (B = 1.02, SE = 1.52, *p* = 0.50). Having a previous MH diagnosis was associated with greater inattention later (B = 4.82, SE = 1.16, *p* < 0.001). School format was not associated with inattention (B = 1.24, SE = 1.21, *p* 0.30), nor was the interaction with MH history (B = 2.55, SE = 2.52, *p* = 0.31) (Fig. 2; Appendix Table 4).

##### Hyperactivity

Sex (B = -6.72, SE = 1.06, *p* < 0.001), ethnicity (B = -2.70, SE = 1.11, *p* < 0.05), and income (B = 3.01, SE = 1.46, *p* < 0.05) were significant predictors of later hyperactivity. Greater hyperactivity was observed for children identified as males, European ancestry, and from households with lower family income. Having a previous MH diagnosis was associated with greater hyperactivity later (B = 4.51, SE = 1.12, *p* < 0.001. School format was not associated with later hyperactivity (B = 1.17, SE = 1.18, *p* = 0.30), nor was the interaction with MH history Figure 2; Appendix Table 5).

#### High school: Grades 9–12

##### Depression

**Fig. 3:**
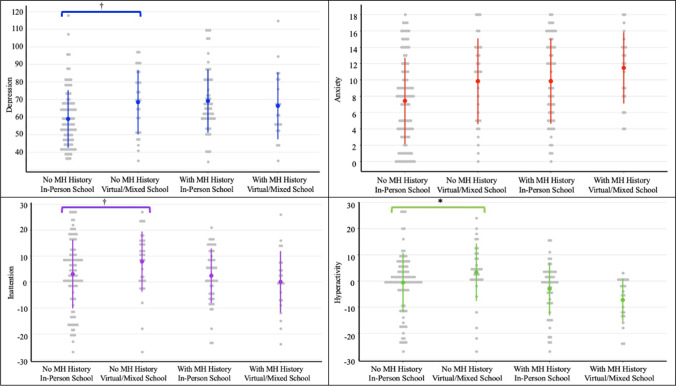
School Format and Mental Health Symptoms Among High School Children (grades 9 to 12) With and Without a History of Pre-COVID Mental Health Problems (*n* = 253). Note. Grey dots represent total values for each MH outcome, with greater values indicating greater MH symptoms (i.e., worse MH). For inattention and hyperactivity, a score of zero represents typical symptom levels. The colored dots represent mean values for each group (MH x School Format) with error bars represented by colored lines. Brackets above groups with “No MH History” identify significant (*p < 0.05) and trending (†*p* < 0.10) interaction effects between school format and pre-pandemic MH history

Greater depression was associated with having a previous MH diagnosis (B = 5.20, SE = 2.55, *p* < 0.05), and attending school virtually (B = -8.18, SE = 3.75, *p* < 0.05). The interaction effect trended towards significance (B = 11.09, SE = 5.77, *p* = 0.06) with results comparing previously healthy children (no MH history) showing greater depression among those who attended school virtually. Sex was a significant predictor of later depression (B = 8.12, SE = 2.53, *p* < 0.01) with greater depression among females. Ethnicity was not associated with depression (B = -2.67, SE – 2.82, *p* = 0.34) (Fig. 3, Appendix Table 6).

##### Anxiety

Greater anxiety was associated with having a previous MH diagnosis (B = 1.72, SE = 0.75, *p* < 0.05) and attending school virtually (B = -1.96, SE = 0.83, *p* < 0.05). Sex (B = 2.36, SE = 0.74, *p* < 0.01) and household income (B = 1.78, SE = 0.88, *p* < 0.05) were associated with later anxiety, with greater anxiety for females and children from lower income households. Ethnicity was not associated with anxiety (B = -1.08, SE = 0.82, = 0.19), nor was the interaction between school format and MH history (Fig. 3, Appendix Table 7).

##### Inattention

Having a previous MH diagnosis (B = -6.89, SE = 3.17, *p* < 0.05) and attending school virtually (B = -5.14, SE = 2.38, *p* < 0.05) were associated with greater inattention. The interaction effect trended towards significance (B = 7.45, SE = 3.80, *p* = 0.05) with results showing that among those without a previous MH diagnosis, those who attended school completely virtually had greater inattention. Sex was a significant predictor of inattention (B = -7.30, SE = 1.73, *p* < 0.001), with greater inattention among males. Ethnicity and household income were not associated with inattention (B = -0.79, SE = 1.97, *p* = 0.66, B = 1.39, SE = 1.97, *p* = 0.59) (Fig. 3; Appendix Table 8).

##### Hyperactivity

After accounting for MH history, attending school virtually was associated with greater hyperactivity (B = -4.03, SE = 2.04, *p* < 0.05). The interaction effect was significant (B = 8.08, SE = 3.29, *p* < 0.05) showing that among those without a previous MH diagnosis, attending school virtually was associated with greater hyperactivity. Sex was associated with later hyperactivity (B = -6.89, SE = 1.51, *p* < 0.001), with greater hyperactivity among males. Ethnicity (B = -1.67, SE = 1.51, *p* = 0.31) and household income (B = 2.08, SE = 1.21, *p* = 0.23) were not associated with hyperactivity. Having a previous MH diagnosis was associated with greater hyperactivity (B = -3.62, SE = 1.54, *p* < 0.05) (Fig. 3, Appendix Table 9).

## Discussion

To date, research suggests a decline in children’s mental health during the pandemic (Cost et al., 2022; Panda et al., 2021; Racine et al., 2021), but further work is needed to understand contributors to this pattern. To the best of our knowledge, this is the first study to offer insights into the school contexts of in-person and virtual learning according to parents and their children. Recent research has offered evidence that attending school virtually is related to worse mental health (Verlenden et al., 2021), but aspects of the school environment, such as learning activities and public health measures, have not been explored. This study examined the experiences of families and their children attending school in Ontario, Canada, during the pandemic. Moreover, we examined whether school format was associated with child and youth mental health symptoms differently among elementary and high school aged children. We presented results suggesting that the in-person learning context during the pandemic included a significant focus on public health measures, with less engagement in social learning activities. Reports from parents and their children illustrated that the in-person school environment was much different than it was prior to the pandemic. With children spending most of their time each week in school, it is important to consider a change to this environment as a potential contributor to their mental health and wellbeing (Jerusalem & Hessling, 2009; O’Connor et al., 2019; Shochet et al., 2006; Riekie et al., 2017).

### School experiences during the pandemic: What are children and youth doing at school?

When children were attending school in-person, they demonstrated high adherence to public health safety measures to prevent the spread of COVID-19. In contrast, children did not engage in social and supportive learning activities (group work; 1:1 time with teachers) regularly. Indeed, at school, children were directed to avoid socializing with others as part of the public health instructions to schools (Ministry of Education, 2020). Implementation of these measures (Government of Ontario’s Guide to Reopening Schools, 2020) are concerning given decades of pre-pandemic research highlighting the benefits of socializing for mental health (Loades et al., 2020; Riekie et al., 2017; Valiente et al., 2020; Vollet et al., 2017) and as such, these measures have potentially created structural barriers to students’ mental well-being. For instance, wearing masks has been identified as a barrier to social interactions (Carbon, 2020), due to greater difficulty reading facial expressions and emotions. While Carbon’s sample was with adults, young people are likely to experience even greater difficulty reading emotions and social cues necessary for social interactions while at school.

Research on the socialization processes in children’s learning at school has illustrated that engaging with peers and teachers in the classroom offers opportunities to develop social and emotional skills, which are in turn correlated with academic success (Slavin, 2013; Valiente et al., 2020). Moreover, studies from fields of education and psychology provide evidence that having at least one positive relationship at school, either with a peer or a teacher, is associated with behavioural and emotional wellbeing for children (Valiente et al., 2020; Vollet et al., 2017). Opportunities to engage with peers during learning activities or extracurriculars can support the development of cooperative skills that support cognitive and socio-emotional achievement as they require children to share and communicate ideas while listening to comprehend the perspectives of others (Slavin, 2013; Valiente et al., 2020). Beyond peer socialization, student–teacher relationships are known to support academic achievement, but also children’s sense of autonomy and belonging in the classroom (Reeve, 2016), which are additional correlates of achievement (Marshik et al., 2017; Vollet et al., 2017).

Although over 70% of parents in this study opted for their child to attend school in-person when available, schools in Ontario were closed to in-person learning for lengthy periods of the school year, requiring all children to attend school virtually. While schools made good efforts to provide children with resources that would allow them to learn from home (i.e., providing electronic devices), our results presented evidence of some barriers to learning remotely. Among all school-aged children (grades K-12), discrepancies between parent and child reports were found for access to supervision while attending school virtually. Parents of younger children (K-8) reported being less able to supervise their children, while the opposite was true for high school, with youth reports indicating less access to available support when attending school virtually. One explanation for these findings is that parental engagement may be lower for older children. As children age, parental involvement in schoolwork declines due to an increase in child autonomy, however, parental involvement in high school is an important indicator of mental health (Wang & Sheikh-Khalil, 2014). Our results are aligned with a recent qualitative study about virtual learning from home during the pandemic, noting that some parents had trouble balancing their own responsibilities with supporting their children’s schooling (Lee, 2020). We also found discrepant reports from parents and high-school aged children regarding access to a quiet and distraction-free space when attending school virtually. In high school, almost one-third of child reports, (31.7%) noted that they did not have a distraction-free, quiet space for learning. As all school format choices in high school involved virtual learning, a portion of youth were required to attend school for at least part of their week in a space they felt was not conducive to their learning. Together, these results suggest that learning from home was more difficult than learning at school for both parents and children.

### School format and mental health

The findings from the current study are consistent with growing research demonstrating the deterioration of children’s mental health during the pandemic (Cost et al., 2022; de Miranda et al., 2020; Lee, 2020; Panda et al., 2021; Racine et al., 2021; Verlenden et al., 2021). Overall, youth in this study experienced significant levels of depression, anxiety, inattention, and hyperactivity, irrespective of school format. One explanation for this might be that our measure of school format did not consider school factors beyond modality that may directly impact mental health. For example, ample research has shown that factors such as academic achievement (O’Connor et al., 2019), competency beliefs (Jerusalem & Hessling, 2009; Marshik et al., 2017), engagement (Vollet et al., 2017), belonging (Marshik et al., 2017) and home-school engagement (Rueger et al., 2014) support student wellbeing, though our current analyses did not account for these non-academic factors.

Results showed that children’s mental health history was the largest contributor to their future mental health, which is supported by previous research (Cost et al., 2022; Golberstein et al., 2020). Children with a pre-pandemic mental health diagnosis experienced the greatest symptom burden. This finding was consistent across all mental health outcomes for both elementary and high school analyses. Results further indicated that school format is an important contributor to mental health outcomes for some students (Appendix Tables 10 and 11). Among high school students, attending school completely virtually was associated with worse mental health outcomes across all domains examined, even after accounting for mental health history and demographic factors. These findings are consistent with those of a recent study showing that increased screen use, including e-learning, was associated with worse mental health symptoms among school-aged children and youth (Li et al., 2021).

It is important to note that our analyses tested for an interaction between mental health history and school format and this interaction reached significance for hyperactivity, but trended towards significance (*p* < 0.10) for all other mental health outcomes. When assessing the role of school format within mental health groupings, there was evidence to suggest that among those without a previous mental health diagnosis, attending school in-person at least some of the time offered some protective effect on mental health symptoms. While interaction effects often trended towards significance (*p*-value range from 0.05 -0.07), the variation across mental health outcomes was large, which is known to reduce statistical power (Norton & Strube, 2001). Among children without a reported mental health diagnosis before the pandemic, there were nonetheless some individuals with clinically significant levels of depression and anxiety pre-pandemic. We chose not to remove these individuals, despite the variance within groups, so that the sample was representative of children and youth with all mental health histories during the pandemic.

It is also noteworthy that among individuals without a previous mental health diagnosis, those who attended school virtually experienced mental health symptoms at comparable levels to those who had been struggling with a mental health diagnosis even prior to the pandemic. High school students with a pre-pandemic mental health diagnosis had clinical levels of anxiety and depression, regardless of school format. It is possible that those with unrecognized pre-pandemic social anxiety may have preferred virtual learning (Cost et al., 2022) . Learning virtually from home may also have the added benefit of familial support (Roy et al., 2022) or reduced worry about contracting the virus (Larsen et al., 2022). More interesting was that similar symptom levels were found for those without a previous mental health diagnosis who attended school virtually, compared to peers with a pre-pandemic mental health diagnosis. Meanwhile, more virtual learning in high school was associated with significantly greater mental health symptoms in high school, meaning that attending school even partially in-person (i.e., mixed format) was associated with fewer symptoms.

Among elementary and middle school aged children, school format trended towards significance with respect to association with mental health symptoms. It is critical to interpret these results in the context of how the pandemic has indirectly impacted the school environment. Earlier we discussed how a child’s development is impacted by their relationships with surrounding environments (Bronfenbrenner & Morris, 1998), with school being of great interest. Our descriptive results show that in-person learning involved high levels of engagement with public health measures and rare or nil experiences with social and supportive learning activities, resulting in a school environment that looked very different to children than it did prior to the pandemic. Indeed, many of the aspects of school that support children’s mental health were limited. While beyond the scope of this study, extracurricular activities outside of the classroom context were also disrupted (Government of Ontario’s Guide to Reopening Schools, 2020; Ministry of Education, 2020), further preventing youth from engaging in socialization that supports their mental health. Therefore, it is possible that simply opening schools to in-person learning was insufficient, as many key activities were altered or absent. In addition, for children with pre-existing mental health vulnerability, the short periods of school re-opening during the 2020–2021 academic year may not have been sufficient to buffer the effects of virtual learning on children’s mental health. Thus, it may be that children with greater mental health risk were not exposed to a sufficient “dose” of in-person learning to experience a protective effect.

There is evidence from pre-pandemic research showing that schools are an important space for offering informal or non-clinical supports for children’s mental health (de Miranda et al., 2020), but the school closures limit children’s access to such supports. Repeated periods of school closures and re-openings may also have had a negative impact on children’s mental health due to the environmental, social, and academic instability (Patel, 2020). Structure and routine are especially important for children with increased mental health risk (Georgiades et al., 2019). Children attending school in-person may have experienced increased mental health symptoms (e.g. anxiety) in the school environment compared with previously healthy children, due to COVID-19-related health risks (Cost et al., 2022), or repeated school-closures. Research has also reported loss of MH services among clinical populations during times of school closures (Allison & Levac, 2022; Charalampopoulou et al., 2022), which may have elevated the MH risk for children in our sample, especially those from clinical cohorts. This was beyond the scope of the current study, but should be considered when interpreting the findings. Both COVID-19-related stress and service loss could have nullified the potential benefits of in-person school experienced by their healthy counterparts.

Before the pandemic, schools provided not only formal mental health services for students (Allison & Levac, 2022), but also informal supports, by creating a space where children can learn more about themselves (Jerusalem and Hessling, 2009; Riekie et al., 2017) and form relationships with their peers and school staff (Marshik et al., 2017; Reeve, 2016; Shochet et al., 2006; Vollet et al., 2017). Pre-pandemic, these informal supports may have been sufficient to support the mental health and wellbeing of some children. It is possible that even during periods of school re-openings, barriers to such supports were still in effect in order to reduce the spread of COVID-19 (Government of Ontario’s Guide to Reopening Schools, 2020; Ministry of Education, 2020; Georgiades et al., 2019). As the vast majority of children in Canada (80%; Golberstein et al., 2020) did not have a mental health problem pre-pandemic, the association of virtual school in high school, with effects trending towards significance replicated in younger grades is an important one, especially since findings appear to be driven by worsening mental health among children without a previous mental health diagnosis. Based on these results, we suggest that the enforcement of virtual schooling may be a salient contributor to the magnitude of the global worsening of children’s mental health, as observed in a recent meta-analysis (Racine et al., 2021), which is otherwise poorly understood (Golberstein et al., 2021). Additional components of children’s school environments that were disrupted by the pandemic should be investigated as potential contributors to this decline in mental health and well-being amongst children and youth.

### Strengths and limitations

To the best of our knowledge, this is the only empirical study that has investigated the role of school format (i.e., in-person, virtual, mixed in-person and virtual) on mental health outcomes during the pandemic for children with and without a pre-existing mental health diagnosis. While commentaries and opinion pieces have theorized about the potential impacts of the changes to education for children (Masonbrink & Hurley, 2020; Patel, 2020), examination of school experiences from the perspectives of children, youth, and their families is lacking (Ravens-Sieberer et al., 2022).

The present study included a large sample that was comprised of children across all school ages from kindergarten to grade 12. Findings from our study revealed that the school format offerings differed by grade level and analyses offered new descriptive information about learning activities and engagement in public health measures while attending school during the pandemic. The longitudinal design, with baseline in May 2020, also allowed us to report on differences in mental health outcomes (February/March 2021), comparing children with and without a pre-existing mental health diagnosis. Using validated measures for child mental health symptoms, we were able to test associations between school format and future mental health while accounting for children’s mental health history prior to the pandemic.

While the above are strengths of this study, there are known limitations to address. While some families opted for virtual learning during the pandemic, there were times when all children were forced to attend school virtually. Due to phases of school closures and re-opening during the time of data collection (Government of Ontario’s Guide to Reopening Schools, 2020), it is possible that some children had experienced virtual learning, despite having opted for in-person learning. As a result, some children may have experienced both in-person and virtual modes of learning. However, our survey asks parents and their children to report on the current mode of learning when schools were open to in-person learning to help account for this.

By design, the project utilized established cohorts, including two with clinical populations. We reported just under one third of children had a pre-pandemic mental health diagnosis, which is higher than the general population prevalence rate. One of the goals of the project was to better understand the impact of the pandemic for children with and without mental health problems and so we wanted to ensure we had good representation of both groups. While this limits our ability to generalize our results to larger community samples, our study offers new information about the school experiences of children with a mental health diagnosis during the pandemic.

Our measure of school format and the school context are novel, but as discussed above, our results using this measure align with previous research. More importantly, it was necessary to develop such a tool in order to capture the school context during the pandemic, since no other appropriate tool exists. While biases exist with self-reports (Fulmer & Frijters, 2009), and it is possible that parents supported children when completing the surveys, including both youth and parent reports addressed a critical gap in the literature. Most related studies have used only parent reports (e.g., Verlenden et al., 2021) which leaves the perspectives of children less understood. This is especially problematic for reports about the school setting since parents are likely less accurate about the activities that happen at school, which may help to explain the discrepancies between child and parent reports in this study and others (Curhan et al., 2020; Schwab et al., 2020). It is also noteworthy that our sample underrepresents ethnic minorities and children from lower-income households. While our results showed that both child ethnicity and household income were associated with children’s mental health, future studies should further investigate socio-demographic factors as contributors to child mental health in the context of the pandemic. Research on social determinants of health, including race, ethnicity, household income, have reported on disproportionate levels of poor mental health among minority and lower income communities (Bernardini et al., 2021). Such inequities must be further explored, especially in households with children and youth who may be experiencing elevated levels of mental health symptoms during the pandemic.

## Conclusion

This study finds that in-person school involved high adherence to public health measures and few occurrences of social learning activities that are known to support not just achievement (Marshik et al., 2017; O’Connor et al., 2019; Reeve, 2016; Slavin, 2013; Valiente et al., 2020), but also mental health (Shochet et al., 2006). In addition, virtual school attendance was associated with worse mental health, especially in high school. Adolescents with no pre-pandemic mental health risk may be especially at-risk when regular in-person school activities are limited. For some children, school may be the primary source of support for mental health. Understanding how schools informally support youth mental health is critical, so that similar support can be offered beyond the classroom. Extending informal supports beyond the classroom may look like increased opportunities for youth to socialize and engage in cooperative practices with peers outside of school. However, connecting with teachers, in addition to peers, is also important (Vollet et al., 2017). Efforts to strengthen home-to-school communications are also recommended, especially during high school, when families may become less involved in children’s school experiences (Wang & Sheikh‐Khalil, 2014).

Our results demonstrate that simply opening school buildings to in-person learning is not sufficient to support children’s mental health. Efforts must be made to also provide opportunities for enablers of good mental health, such as social learning, increased peer and teacher interactions, and extracurricular activities. These were aspects of school that were limited during the pandemic, as shown in our study and others (Masonbrink & Hurley, 2020; Panda et al., 2021; Verlenden et al., 2021). This work provides empirical support for the call to action for a change to in-person learning during and beyond the pandemic.

## Data Availability

The data that support the findings of this study are not openly available and are available from the corresponding author upon reasonable request (including a study outline), subject to review.

## Appendix

**Table 2:** RCADS: Grades 4–8

Block 1	B	t	LLCI	ULCI
Intercept	**63.67 (1.34)**	**47.52*****	**61.03**	**66.30**
Sex	**-5.47 (1.68)**	**-3.26****	**-8.76**	**-2.17**
Ethnicity	**-3.59 (1.73)**	**-2.07***	**-6.99**	**-.19**
Income	3.74 (2.14)	1.74	-.48	7.97
Block 2				
Intercept	**59.31 (1.54)**	**38.49*****	**56.28**	**62.34**
Sex	**-5.24 (1.63)**	**-3.22****	**-8.43**	**-2.04**
Ethnicity	-2.01 (1.72)	-1.17	-5.39	1.37
Income	3.59 (2.10)	1.71†	-.55	7.72
MH	**9.19 (1.74)**	**5.29*****	**5.78**	**12.60**
School Format (SF)	2.07 (1.83)	1.13	-1.52	5.66
Block 3				
Intercept	**60.06 (1.59)**	**37.73*****	**56.93**	**63.19**
Sex	**-5.38 (1.62)**	**-3.31****	**-8.58**	**-2.19**
Ethnicity	-2.00 (1.71)	-1.17	-5.37	1.37
Income	3.52 (2.09)	1.69†	-.59	7.62
MH	**7.19 (2.05)**	**3.50****	**3.16**	**11.22**
School Format (SF)	-.23 (2.22)	-.10	-4.59	4.13
Interaction (MHxSF)	6.85 (3.77)	1.81†	-.57	14.27

Bolded values indicate significance as follows:^ ***^ *p* < .001, ***p* < .01, * *p* < .05, † *p* < .10

**Table 3:** SCARED: Grades 4–8

Block 1	B	t	LLCI	ULCI
Intercept	**7.50 (.38)**	**19.68******	**6.75**	**8.25**
Sex	-.58 (.48)	-1.21	-1.52	.36
Ethnicity	**-2.04 (.50)**	**-4.11*****	**-3.03**	**-1.07**
Income	.56 (.61)	.92	-.64	1.76
Block 2				
Intercept	**6.03 (.43)**	**13.95*****	**5.18**	**6.88**
Sex	-.51 (.46)	-1.12	-1.41	.38
Ethnicity	**-1.58 (.49)**	**-3.24****	**-2.54**	**-.62**
Income	.48 (.59)	.82	-.68	1.66
MH	**3.02 (.49)**	**6.17*****	**2.06**	**3.99**
School Format (SF)	1.01 (.51)	1.96†	-.00	2.02
Block 3				
Intercept	**5.99 (.45)**	**13.38*****	**5.11**	**6.87**
Sex	-.50 (.46)	-1.10	-1.41	.40
Ethnicity	**-1.58 (.49)**	**-3.24****	**-2.54**	**-.62**
Income	.49 (.59)	.83	-.68	1.66
MH	**3.15 (.58)**	**5.41*****	**2.00**	**4.29**
School Format (SF)	1.15 (.62)	1.83†	-.08	2.37
Interaction (MHxSF)	-.42 (1.07)	-.39	-2.52	1.69

Bolded values indicate significance as follows:^***^ *p* < .001, ***p* < .01, * *p* < .05, † *p* < .10

**Table 4:** Inattention: Grades 4–8

Block 1	B	t	LLCI	ULCI
Intercept	**7.17 (.90)**	**8.00*****	**5.43**	**8.97**
Sex	**-5.80 (1.11)**	**-5.24*****	**-7.98**	**-3.63**
Ethnicity	-2.09 (1.15)	-1.82†	-4.35	.17
Income	.96 (1.45)	.66	-1.89	3.82
Block 2				
Intercept	**4.91 (1.04)**	**4.71*****	**2.86**	**6.95**
Sex	**-5.73 (1.09)**	**-5.26*****	**-7.87**	**-3.59**
Ethnicity	-1.31 (1.15)	-1.14	-3.57	.94
Income	.88 (1.43)	.61	-1.95	3.71
MH	**4.82 (1.16)**	**4.15*****	**2.53**	**7.10**
School Format (SF)	1.28 (1.21)	1.06	-1.10	3.66
Block 3				
Intercept	**5.20 (1.08)**	**4.81*****	**3.07**	**7.32**
Sex	**-5.80 (1.09)**	**-5.31*****	**-7.95**	**-3.65**
Ethnicity	-1.31 (1.15)	-1.14	-3.57	.95
Income	.85 (1.43)	.59	-1.98	3.67
MH	**4.04 (1.39)**	**2.91****	**1.31**	**6.78**
School Format (SF)	.42 (1.47)	.28	-2.48	3.31
Interaction (MHxSF)	2.56 (2.52)	1.01	-2.40	7.51

Bolded values indicate significance as follows:^***^ *p* < .001, ***p* < .01, * *p* < .05, † *p* < .10

**Table 5:** Hyperactivity: Grades 4–8

Block 1	B	t	LLCI	ULCI
Intercept	**4.93 (.87)**	**5.69*****	**3.23**	**6.64**
Sex	**-6.67 (1.06)**	**-6.31*****	**-8.81**	**-4.63**
Ethnicity	**-2.70 (1.11)**	**-2.43***	**-4.88**	**-.52**
Income	**3.01 (1.46)**	**2.07***	**.13**	**5.88**
Block 2				
Intercept	**2.80 (1.01)**	**2.78****	**.82**	**4.78**
Sex	**-6.65 (1.05)**	-**6.35*****	**-8.71**	**-4.59**
Ethnicity	-1.96 (1.11)	-1.77†	-4.15	.22
Income	**2.91 (1.45)**	**4.57***	**.06**	**5.76**
MH	**4.51 (1.12)**	**4.04*****	**2.32**	**6.71**
School Format (SF)	1.17 (1.18)	.99	-1.15	3.49
Block 3				
Intercept	**2.81 (1.05)**	**2.69****	**.75**	**4.88**
Sex	**-6.65 (1.05)**	**-6.33*****	**-8.72**	**-4.59**
Ethnicity	-1.96 (1.11)	-1.77†	-4.15	.22
Income	**2.91 (1.45)**	**2.01***	**.05**	**5.76**
MH	**4.47 (1.34)**	**3.34*****	**1.84**	**7.10**
School Format (SF)	1.13 (1.44)	.78	-1.71	3.97
Interaction (MHxSF)	.13 (2.43)	.05	-4.65	4.91

Bolded values indicate significance as follows:^***^ *p* < .001, ***p* < .01, * *p* < .05, † *p* < .10

**Table 6:** RCADS: High School 9–12

Block 1	B	t	LLCI	ULCI
Intercept	**58.71 (2.17)**	**27.10*****	**54.44**	**62.99**
Sex	**8.12 (2.53)**	**3.21****	**3.13**	**13.11**
Ethnicity	-2.67 (2.82)	-.95	-8.24	2.89
Income	5.15 (3.00)	1.71†	-.79	11.08
Block 2				
Intercept	**60.11 (3.34)**	**18.02*****	**53.53**	**66.70**
Sex	**6.97 (2.55)**	**2.73****	**1.94**	**12.01**
Ethnicity	-2.81 (2.78)	-1.01	-8.30	2.68
Income	4.59 (2.99)	1.54	-1.31	10.50
MH	**5.20 (2.55)**	**2.04***	**.16**	**10.24**
School Format (SF)	-3.49 (2.90)	-1.21	-9.21	2.23
Block 3				
Intercept	**63.72 (3.80)**	**16.78*****	**56.23**	**71.21**
Sex	**6.72 (2.54)**	**2.65****	**1.72**	**11.73**
Ethnicity	-2.43 (2.78)	-.87	-7.91	3.06
Income	4.59 (2.96)	1.55	-1.27	10.44
MH	-3.00 (4.96)	-.60	-12.77	6.79
School Format (SF)	**-8.18 (3.75)**	**-2.18***	**-15.59**	**-.77**
Interaction (MHxSF)	11.09 (5.77)	1.92†	-.19	22.47

Bolded values indicate significance as follows:^***^ *p* < .001, ***p* < .01, * *p* < .05, † *p* < .10

**Table 7:** SCARED: High School 9–12

Block 1	B	t	LLCI	ULCI
Intercept	**7.46 (.62)**	**12.00*****	**6.23**	**8.69**
Sex	**2.36 (.74)**	**3.18****	**.90**	**3.83**
Ethnicity	-1.08 (.82)	-1.08	-2.71	.55
Income	**1.78 (.88)**	**2.03***	**.06**	**3.51**
Block 2				
Intercept	**8.39 (.93)**	**9.19*****	**6.75**	**10.43**
Sex	**1.95 (.74)**	**2.64****	**.49**	**3.41**
Ethnicity	-1.18 (.81)	-1.46	-2.77	.42
Income	1.55 (.86)	1.81†	-.14	3.25
MH	**1.72 (.75)**	**2.29***	**.24**	**3.19**
School Format (SF)	**-1.96 (.83)**	**-2.35***	**-3.60**	**-.31**
Block 3				
Intercept	**8.79 (1.06)**	**8.27*****	**6.69**	**10.88**
Sex	**1.95 (.74)**	**2.63****	**.49**	**3.42**
Ethnicity	-1.16 (.81)	1.43	-2.76	.44
Income	1.55 (.86)	1.80†	-.15	3.24
MH	1.22 (1.45)	.84	-1.64	4.08
School Format (SF)	**-2.22 (1.06)**	**-2.09***	**-4.32**	**-.12**
Interaction (MHxSF)	.68 (1.69)	.40	-2.66	4.01

Bolded values indicate significance as follows:^***^ *p* < .001, ***p* < .01, * *p* < .05, † *p* < .10

**Table 8:** Inattention: High School 9–12

Block 1	B	t	LLCI	ULCI
Intercept	**7.09 (1.47)**	**4.83*****	**4.19**	**9.98**
Sex	**-7.30 (1.73)**	**-4.22*****	**-10.72**	**-3.89**
Ethnicity	-.79 (1.88)	-.42	-4.51	2.93
Income	1.39 (1.97)	.71	-2.49	5.28
Block 2				
Intercept	**9.31 (2.14)**	**4.04*****	**5.08**	**13.53**
Sex	**-7.08 (1.76)**	**-4.98*****	**-10.56**	**-3.61**
Ethnicity	-.87 (1.89)	-.46	-4.60	2.86
Income	1.19 (1.99)	.60	-2.74	5.11
MH	-1.73 (1.77)	-.98	-5.23	1.76
School Format (SF)	-2.28 (1.89)	-1.21	-6.00	1.44
Block 3				
Intercept	**11.39 (3.39)**	**4.79*****	**6.70**	**16.08**
Sex	**-7.20 (1.75)**	**-4.12*****	**-10.66**	**-3.75**
Ethnicity	-.68 (1.88)	-.36	-4.39	3.04
Income	1.12 (1.96)	.57	-2.75	4.99
MH	**-6.89 (3.17)**	**-2.18***	**-13.14**	**-.65**
School Format (SF)	**-5.14 (2.38)**	**-2.16***	**-9.83**	**-.45**
Interaction (MHxSF)	7.45 (3.80)	1.96†	-.05	14.94

Bolded values indicate significance as follows:^***^ *p* < .001, ***p* < .01, * *p* < .05, † *p* < .10

**Table 9:** Hyperactivity: High School 9–12

Block 1	B	t	LLCI	ULCI
Intercept	**2.22 (1.28)**	**1.74***	**-.30**	**4.74**
Sex	**-6.89 (1.51)**	**-4.57*****	**-9.87**	**-3.91**
Ethnicity	-1.67 (1.65)	-1.01	-4.93	1.59
Income	2.08 (1.71)	1.21	-1.31	5.46
Block 2				
Intercept	**3.87 (1.86)**	**2.08***	**.20**	**7.54**
Sex	**-6.26 (1.52)**	**-4.11*****	**-9.26**	**-3.25**
Ethnicity	-1.69 (1.63)	-1.03	-4.93	1.54
Income	2.06 (1.72)	1.20	-1.34	5.46
MH	**-3.62 (1.54)**	**-2.35***	**-6.65**	**-.58**
School Format (SF)	-.93 (1.63)	-.57	-4.15	2.30
Block 3				
Intercept	**6.13 (2.04)**	**3.01****	**2.11**	**10.15**
Sex	**-6.38 (1.50)**	**-4.23*****	**-9.34**	**-3.41**
Ethnicity	-1.49 (1.62)	-.92	-4.70	1.71
Income	2.00 (1.70)	1.18	-1.35	5.36
MH	**-9.22 (2.74)**	**-3.36*****	**-14.64**	**-3.81**
School Format (SF)	**-4.03 (2.04)**	**-1.98***	**-8.05**	**-.02**
Interaction (MHxSF)	**8.08 (3.29)**	**2.45***	**1.58**	**14.58**

Bolded values indicate significance as follows:^***^ *p* < .001, ***p* < .01, * *p* < .05, † *p* < .10

**Table 10:** Mental Health Outcomes Among Elementary (K-8) School Children By School Format

	Whole Group	In-Person School Format	Virtual or Mixed School Format
	M (SD)	M (SD)	M (SD)
Depression (RCADS; *n* = 650)	60.30 (18.05)	59.77 (17.44)	61.40 (19.61)
Anxiety (SCARED; *n* = 663)	6.57 (5.22)	6.33 (5.08)	6.97 (5.43)
Inattention (SWAN; *n* = 657)	3.49 (11.06)	2.96 (10.97)	4.09 (11.24)
Hyperactivity (SWAN; *n* = 657)	1.61 (10.99)	1.14 (10.50)	2.16 (11.83)

T-score reported for Depression (RCADS); Total scores reported for all other measures of youth mental health (SCARED, TIDES, SWAN). The SCARED score represents the Generalized Anxiety Disorder subscale (GAD) with scores of 9 or greater indicating clinically significant anxiety. SWAN ranges from -27 to + 27; all others are in positive range

**Table 11:** Mental Health Outcomes Among High School (Grades 9–12) Children By School Format

	Whole Group	Virtual School Format	Mixed School Format
	M (SD)	M (SD)	M (SD)
Depression (RCADS; *n* = 197)	63.94 (17.79)	68.26 (18.43)	62.48 (17.91)
Anxiety (SCARED; *n* = 188)	8.94 (5.35)	10.55 (4.97)	8.05 (5.37)
Inattention (SWAN; *n* = 197)	.39 (12.28)	-.84 (12.22)	2.88 (12.38)
Hyperactivity (SWAN; *n* = 197)	-1.31 (10.90)	-.84 (11.23)	-1.46 (10.94)

T-score reported for Depression (RCADS); Total scores reported for all other measures of youth mental health (SCARED, SWAN). The SCARED score represents the Generalized Anxiety Disorder subscale (GAD) with scores of 9 or greater indicating clinically significant anxiety. SWAN ranges from -27 to + 27; all others are in positive range
